# Seed targeted RNAi-mediated silencing of *GmMIPS1* limits phytate accumulation and improves mineral bioavailability in soybean

**DOI:** 10.1038/s41598-019-44255-7

**Published:** 2019-05-23

**Authors:** Awadhesh Kumar, Varun Kumar, Veda Krishnan, Alkesh Hada, Ashish Marathe, Parameswaran C., Monica Jolly, Archana Sachdev

**Affiliations:** 1 Division of Biochemistry, ICAR-Indian Agricultural Research Institute, New Delhi, 110 012 India; 2 Division of Crop Physiology and Biochemistry, ICAR-National Rice Research Institute, Cuttack, Odisha India; 3 Department of Biotechnology and Bioinformatics, Jaypee University of Information Technology, Waknaghat, (H.P.) India

**Keywords:** Metabolic engineering, RNAi

## Abstract

Phytic acid (PA), the major phosphorus reserve in soybean seeds (60–80%), is a potent ion chelator, causing deficiencies that leads to malnutrition. Several forward and reverse genetics approaches have ever since been explored to reduce its phytate levels to improve the micronutrient and phosphorous availability. Transgenic technology has met with success by suppressing the expression of the PA biosynthesis-related genes in several crops for manipulating their phytate content. In our study, we targeted the disruption of the expression of *myo-*inositol-3-phosphate synthase (*MIPS1*), the first and the rate limiting enzyme in PA biosynthesis in soybean seeds, by both antisense (AS) and RNAi approaches, using a seed specific promoter, *vicilin*. PCR and Southern analysis revealed stable integration of transgene in the advanced progenies. The transgenic seeds (T_4_) of AS (MS14-28-12-29-3-5) and RNAi (MI51-32-22-1-13-6) soybean lines showed 38.75% and 41.34% reduction in phytate levels respectively, compared to non-transgenic (NT) controls without compromised growth and seed development. The electron microscopic examination also revealed reduced globoid crystals in the Protein storage vacoules (PSVs) of mature T_4_ seeds compared to NT seed controls. A significant increase in the contents of Fe^2+^ (15.4%, 21.7%), Zn^2+^ (7.45%, 11.15%) and Ca^2+^ (10.4%, 15.35%) were observed in MS14-28-12-29-3-5 and MI51-32-22-1-13-6 transgenic lines, respectively, compared to NT implicating improved mineral bioavailability. This study signifies proof-of-concept demonstration of seed-specific PA reduction and paves the path towards low phytate soybean through pathway engineering using the new and precise editing tools.

## Introduction

Soybean (*Glycine max* L.) is among the most nutritious and economical foods but has a relatively limited consumption and cultivation, accredited to the presence of high levels of PA (*myo*-inositol hexa*kis*phosphate, IP_6_) accounting to ~2% of the total seed dry weight^1^. PA, a ubiquitous phosphorous reserve amassed in plant seeds sourcing phosphorous, inositol and minerals during germination^2,3^ is an anti-nutritive component with multiple negative charges, which make it an efficient chelator of multivalent cations Fe^2+^, Zn^2+^, Ca^2+^ and Mg^2+ ^^4^. The binding results in insoluble salts limiting the bioavailability of minerals^5–7^, affecting millions of people subsisting on grain based diets, mainly in the developing world^8^. The monogastrics, lacking microbial phytases in their gut are also not able to remove phosphates from the *myo*-inositol ring and hence are incapable of utilizing the phosphorus. The undigested PA excreted as phosphate-rich waste consequently poses threat to the environment by eutrophication of waterways^8^. Therefore, manipulating plant phytate levels by genetic means is suggested to be sustainable solution without dispute^9^.

The first generation of low phytate crops were developed using classical breeding and further the efforts were augmented with the aid of modern technologies involving forward genetics approaches. Mutations that blocked the synthesis or accumulation of PA during seed development could result in 50–95% decreased seed phytate crops with homozygous low phytate alleles (lpa) and increased Pi availability^4,10–13^. However, they also showed reduced seed yield, viability, undesirable morphology and susceptibility to pathogens^14–16^. The reverse genetic approaches in contrast, provided certain advantages over the use of random whole-plant gene mutations, including the tissue specific knockdown of PA, thereby eliminating the negative impact on plant functions. Prevalence of earlier reports have documented the successful genetic manipulation of PA in various crops by using an effective, precise and stable double stranded RNAi technology^2,17,18^ and antisense approach^19^.

The biosynthesis of PA predominantly following the lipid independent pathway, involves multiple enzyme catalyzed reactions with MIPS (myo-inositol-3-phosphate synthase) (EC 5.5.1.4), catalyzing the primary and rate limiting step in the pathway and IPK1 (Inositol 1,3,5,6-pentakisphosphate 2-kinase) the final step^2,9^. Generally the genes involved in the first and last steps of PA biosynthesis not only increase the total P levels but also increase the influx of P from vegetative organs into seeds^20^. *MIPS lpa* mutants showed decreased PA accompanied by a molar increase in free phosphate whereas PA decreased with increase in free phosphate and lower inositol phosphates in the *IPK1* mutants^21,22^. The first committed step catalyzed by MIPS involves the formation of inositol-6-phosphate from glucose 6-phosphate followed by sequential phosphorylation at the remaining five positions of the inositol ring in an ordered manner through various enzymes^23^. The coding sequences of *MIPS* showing high conservation at nucleotide level have been cloned and characterized from a number of plant species, such as *Arabidopsis thaliana*^24^, *Citrus paradisii*^25^, common ice plant^26^, tobacco^27^ and soybean^28^. Moreover, four highly similar *MIPS* ESTs have been identified and isolated from soybean during early stage of seed development^29^ of which *MIPS1* isoform is known to express under the control of a strong, developmentally regulated promoter in immature seeds^30^.

Research in the past has evidenced the potential of RNAi technique to generate Ipa rice^2,18^, wheat^31^ and soybean^22^ by effectively down regulating PA pathway genes. Down regulation of *MIPS* gene expression under CaMV35S promoter using self - complementary hairpin RNA was also carried out in soybean^17^ resulting in a drastic reduction (~94.5%) of phytate content in the developed transgenic lines. Reduction however ensued poor seed development and emergence demonstrating an important correlation between *MIPS* expression and seed development.

Therefore, in this study, we attempted to generate transgenic soybean plants by silencing the *MIPS1* under a seed-specific promoter, *vicilin* through both AS and RNAi approaches. The resulting T_4_ transgenic plants were analyzed at the molecular and biochemical levels, revealing a substantial reduction in PA levels. Further, the agronomic traits of the transgenic soybean plants were compared with non-transgenic controls. The ultrastructure studies by transmission electron microscopy also revealed a distinct reduction in the phytate globoids in these transgenics.

## Materials and Methods

### Plant material and vectors

Soybean (*Glycine max* L. Merr. cv DS-9712) seeds were procured from Division of Genetics, ICAR-IARI, New Delhi, India for genetic transformation.

### Construction of a novel binary vector

A new binary vector was created to generate *MIPS1*ihp and *MIPS1*AS constructs. For this, the 5,539 bp and 8,381 bp fragments were excised from pCW66 and pAKVS vectors, respectively with *Eco*RI and ligated to generate a new recombinant binary vector pCWAK (13920 bp) (Supplementary Fig. 1). The recombinant vector thus obtained contains seed specific *vicilin* promoter with multiple restriction site, plant selectable marker gene (*bar*) under right and left border and also a bacterial selectable marker gene which encodes for kanamycin. The binary vector (pCWAK) was tested by restriction analysis using the combination of *Eco*RI and *Xho*I, restriction enzymes. The restriction digestion with *Eco*RI resulted in two bands viz. pAKVS (8.3 kb) and pCW66 (5.5 kb), while *Xho*I digested pCWAK showed three bands including *bar* gene (500 bp) (Supplementary Fig. 2).

### Designing of *MIPS1* intron hairpin (*ihp*) and antisense construct

**Figure 1:**
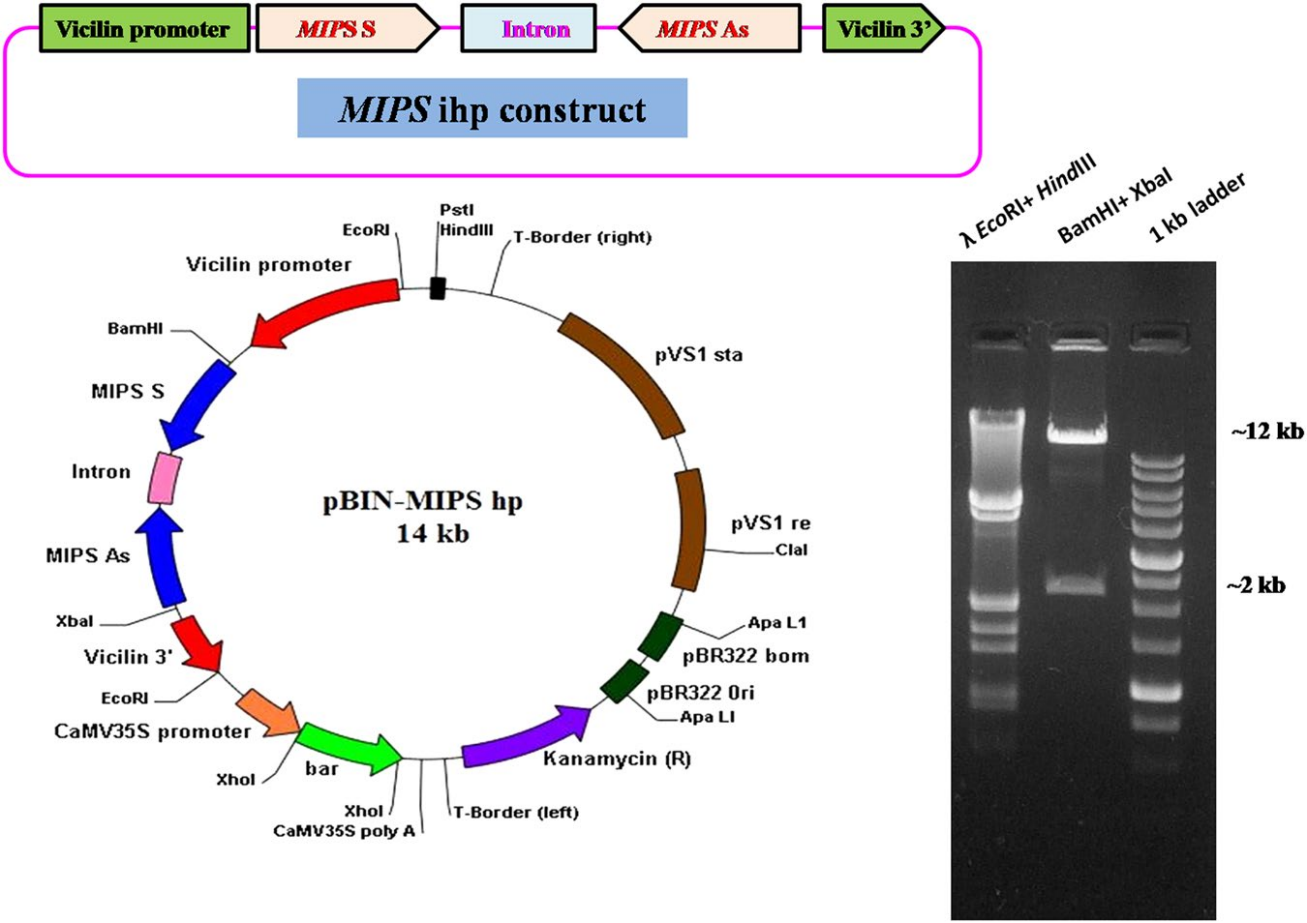
Linear and circular map of pBIN-MIPS ihp construct. Restriction digestion of pBIN-MIPS ihp; Lane1: λ *Eco*RI + *Hin*dIII marker, Lane2: ~12 kb binary vector fragment and ~2 kb *MIPS1 ihp* fragment, Lane3: 1 kb ladder.

To design the *MIPS1* ihp silencing construct, a new binary vector was generated by restriction and ligation of two fragments (5.5 Kb from pCW66 and 8.3 Kb from pAKVS).To facilitate directional cloning of the *MIPS1* sense and antisense fragments, primer pairs of *MIPS1*-ihp cassette were synthesized by inserting restriction sites MIPS *Bam*HI (5′ CGGGATCCCGACCACCGAATCTTGTTCAC 3′), MIPS *Sac*II (5′ TCCCCG CGGGGAAAATCTCAGCCTCATTTC 3′) and MIPS *Hind*III (5′ CCCAAGCTTGGGA CCACCGAACTTGTTCAC 3′), MIPS *Xma*I (5′ TCCCCCCGGGGGGAAAATCTCAG CCTCATTTC 3′) to amplify two 706 bp sequences. An intronic sequence of 396 bp from soybean was amplified with *Sac*II and *Hind*III restriction sites. The amplified *MIPS1* fragments and intronic fragment were cloned separately in the pDrive vector. Thereafter, sense and antisense fragments of *MIPS1* were excised and attached each side with intronic fragment to generate the *MIPS1* ihp cassette (pDrive-MIPS ihp). *MIPS1 ihp* cassette (~1.8 kb) was released with *Bam*HI/*Xba*I from pDrive*-MIPS* ihp and ligated at same site under *vicilin* promoter in the binary vector pCWAK to generate *MIPS1hp* construct **(**Fig. 1**)**. The recombinant clone (~14 kb) containing the *MIPS1* hairpin cassette was screened by restriction analysis and named as pBIN-MIPS hp.

**Figure 2:**
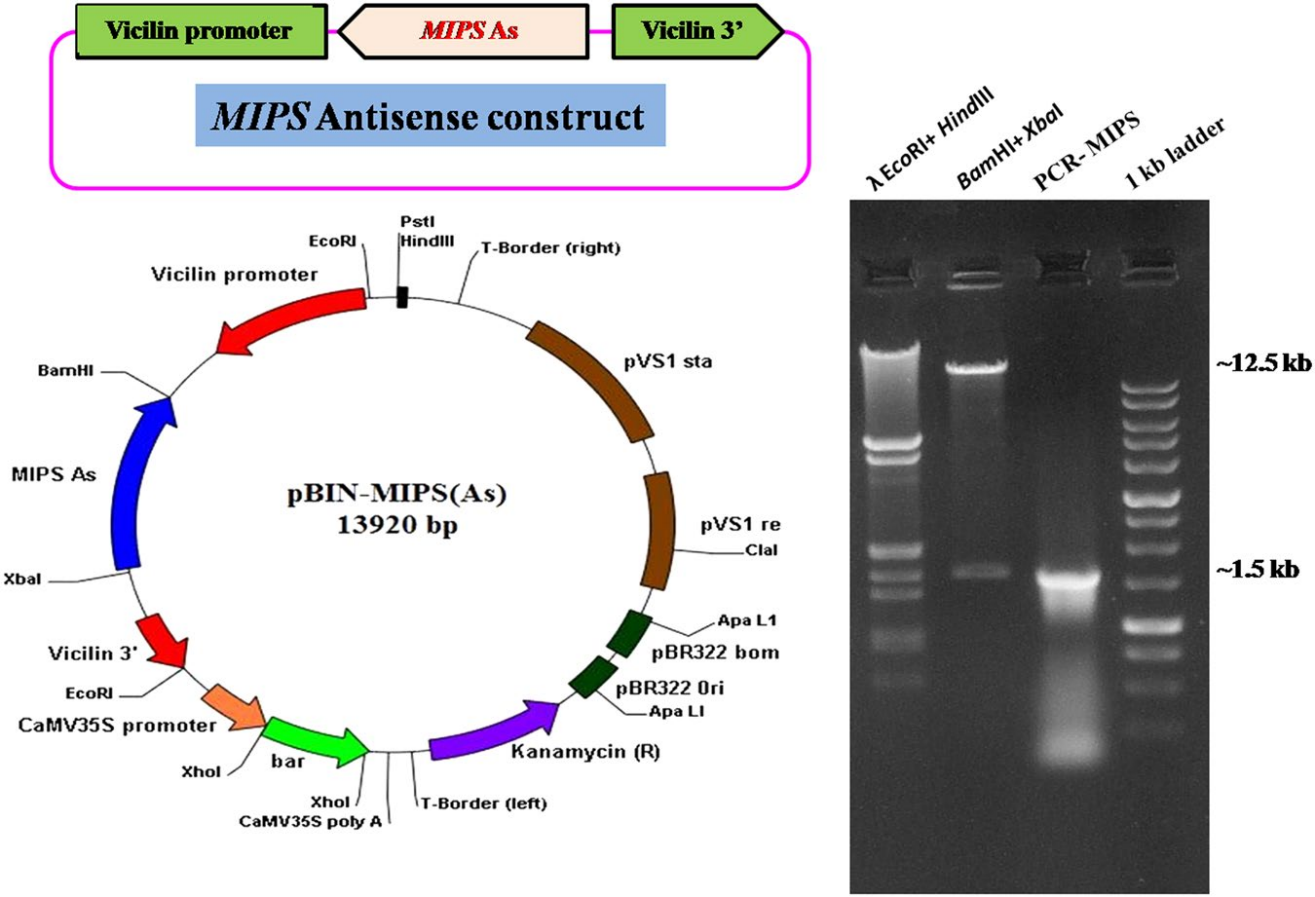
Linear and circular map of pBIN-MIPS AS. *MIPS1* ORF (~1.5 kb) region introduced in antisense orientation under *vicilin* promoter at *Bam*HI/*Xba*I site. Restriction digestion of pBIN-MIPSAS; Lane1: λ-EcoRI/*Hin*dIII marker, Lane2: *Bam*HI/*Xba*I restriction showing ~12.5 kb binary vector fragment and ~1.5 kb *MIPS1* antisense fragment, Lane3: PCR amplification of *MIPS1* from pBIN-MIPS AS, Lane4: 1 kb ladder.

For developing *MIPS1* AS construct, 1.5 kb ORF fragment was amplified using gene specific primers and cloned in pDrive vector to yield pDrive-MIPSAs. The desired orientation was confirmed by *Pst*I and *Eco*RV restriction. The *MIPS1* fragment from pDrive-MIPS was excised and sub-cloned at *Bam*HI/*Xba*I sites in the binary vector pCWAK in an antisense orientation with respect to the *vicilin* promoter and named as pBIN-MIPS (As) (Fig. 2). The positive clones carrying the binary construct (~14 kb) were screened by restriction analysis and by PCR confirmation using specific primers.

### Plant transformation

**Figure 3:**
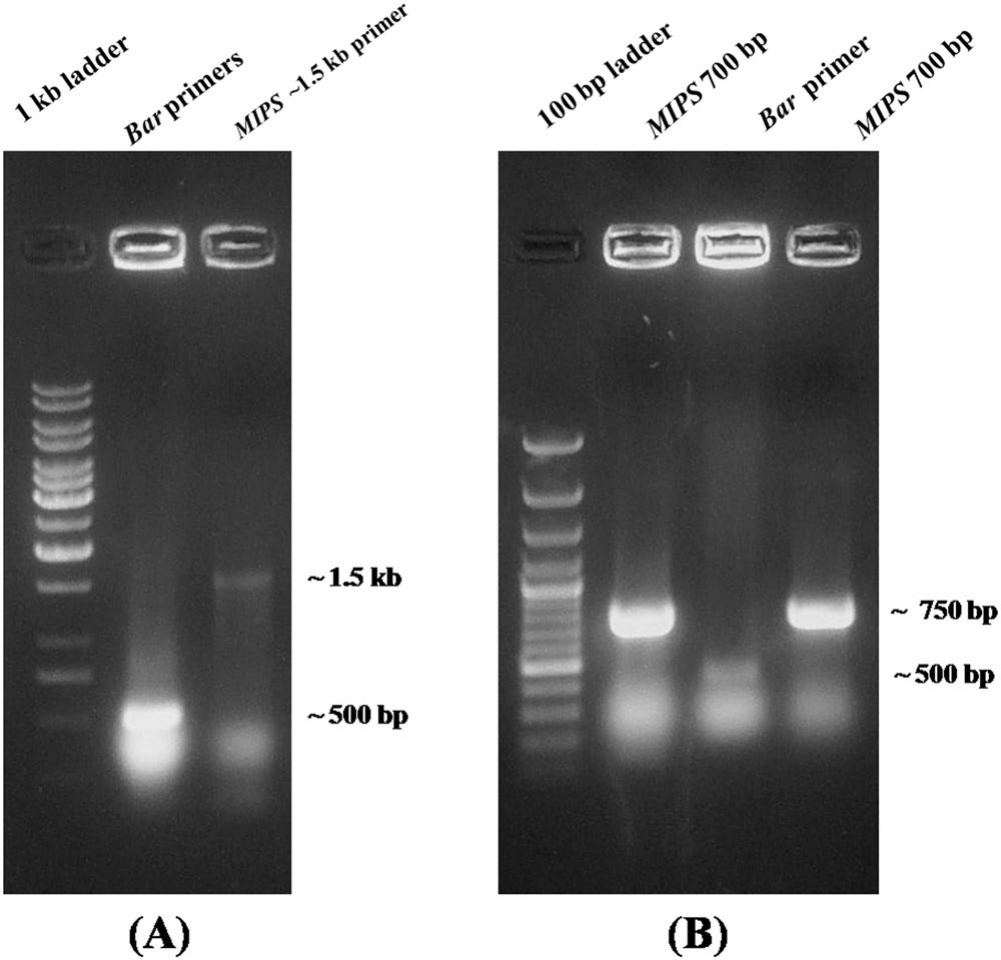
Colony PCR for screening positive colonies harboring constructs. (**A**) Colony PCR of *Agrobacterium* with Bar and MIPSAS specific primers showing ~500 bp bar gene and ~1.5 kb MIPS gene amplification. (**B**) Colony PCR of *Agrobacterium* with bar and MIPS ihp specific primers showing ~500 bp bar gene and ~750 bp MIPS fragments amplification.

The binary vector harboring pBin-MIPShp and pBin-MIPSAs constructs were used for subsequent *Agrobacterium*-mediated transformation of soybean cv. DS9712 cotyledons. *Agrobacterium* colonies containing gene constructs were identified by colony PCR using *bar* and *MIPS1* gene specific primers, which gave expected amplicons (Fig. 3).The *MIPS1* AS and ihp constructs were mobilized into *Agrobacterium tumefaciens* strain EHA105 via triparental mating using PRK2013 as the helper strain. Agrobacterium colonies harboring the recombinant binary constructs were confirmed through colony PCR using bar gene specific primers (FP- 5′GAACGACGCCCGGCCGACAT3′/RP- 5′GTCCAGCTGC CAGAAACCCAC 3′) to obtain an amplicon of ~500 bp. *Agrobacterium* cells (transformed with the pBin-MIPS hp and pBin-MIPS As binary vectors) were used to infect cotyledonary nodes of soybean. Briefly, explants were prepared from 6-days-old seedlings of soybean cultivar DS-9712 by removing the majority of the hypocotyl from the cotyledons and wounding the axillary meristematic tissue at the cotyledonary-node. Seedlings grown in germination medium for explants preparation, Cotyledons were separated and prepared for *Agrobacterium* infection, Explants were inoculated with *Agrobacterium tumefaciens* in co-cultivation medium and kept for 3 days in dark. After 3 days, the cotyledonary nodes embedded into shoot induction medium (SIM) containing 8 mg/L glufosinate to stimulate *de novo* shoot formation from axillary meristematic tissue. After 8–10 days, non transforming explants showed symptom of necrosis due to glufosinate. Selected explants (survived in SIM) transferred into shoot elongation medium (SEM) containing 2 mg/L glufosinate for 12 days and sub-cultured for 12 days in SEM-II. Elongated shoots placed into rooting medium (RM) without glufosinate for 15 days. When roots developed properly, the plantlets were hardened in sterilized pot mix (Agro-peat, Vermiculite and River sand @ 1:2:1 ratio) where Pots covered with plastic bags (with pin holes) and closed tightly to retain humidity. After one week, the bags were removed in order to reduce the humidity gradually until plants became acclimatized to the ambient humidity. Thereafter, the plants were transferred to glass house to maturity under a 16/8 h (light/dark) photoperiod and natural light supplemented with 1,000-W high pressure sodium lamps. Temperature- day 30 °C and night 28 °C, Relative humidity- day 85 °C and night 80 °C. All the plants were fertile and were phenotypically normal.

### Screening of putative transgenics

#### Detection of transgenic soybean plants

PCR analysis was carried out for confirming the integration and presence of transgenic cassette. Genomic DNA (gDNA) was extracted from young leaves of T_0_ transformants using plant DNA isolation kit (Geneaid) following the manufacturer’s protocol and *bar* specific primers were used for the confirmation of ~500 bp amplification in the putative primary transformants. Herbicide-resistance in T_0_ transgenic soybean plants were further verified by leaf painting assay which was performed following the procedure described^32^. Effect of glufosinate (basta) were clearly visible by application of a basta solution (glufosinate: 100 mg/L) to the entire plant body and to the upper leaves as well of NT control plants.

#### Southern hybridization

Genomic DNA was isolated from young leaves using CTAB procedure. DNA (10 μg) was digested with 50 U of *Xho*I overnight and electrophoresed on a 0.8% agarose gel. The size fractionated DNA was then transferred to charged nylon membrane, Biodyne (Hybond N+, Amersham Pharmecia Biotech, UK) through wet capillary method^33^. The *bar* gene (PCR product) labelled with α^32^P-dCTP using DecaLabel DNA labeling kit (Fermentas) was used as a probe. Hybridization was carried out for 18–24 h at 65 °C. The membrane was washed and then exposed to X-ray film (XK-5Kodak film).

#### Quantitative RT-PCR analysis

In order to quantify the level of gene silencing in the transgenics, quantitative real-time PCR (qRT PCR) was performed. Total RNA was isolated from the developing seeds using TRIZOL based procedure. The RNA was treated with RNase free DNase I (Fermentas) to remove the potential DNA contamination. First strand cDNA was synthesized using RevertAid^TM^ H Minus First Strand synthesis kit (Thermo Scientific). qRT-PCR analysis of transgenic seeds was performed using PikoReal real time PCR system (Thermo Scientific) to quantify the transcript level in comparison to the non-transformants. To normalize the data, ^ΔΔ^Ct method was followed using *PEPCo* was used as an internal control. 500 ng of total RNA was reverse transcribed using Oligo dT primers for qRT PCR analysis. Using DyNAmo Color Flash SYBR qPCR kit with the following primer pairs: MIPS1 and PEPCO (FP- 5′ATGTTCATCGAGAATTTTAAGGTT3′/RP- 5′ATCACTTGTACTCGAGAATCAT3′). The reaction was performed in triplicates according to the manufacturer’s guide under the following cycling conditions: 94 °C for 2 min followed by 35 cycles of 94 °C for 30 sec, 55 °C for 30 sec and 94 °C for 30 sec.

#### Phytic acid content analysis by HPLC

The T_4_ transgenic and NT control seeds were dried at 37 °C till a constant weight was achieved, dry weights were recorded and the seeds were ground in a Wiley mill equipped with a 20-mesh screen (Thomas Scientific, USA). PA was extracted as described by^34^. Briefly, 500 mg of defatted soybean flour was extracted overnight in 0.78 M HCl. The sample was further sonicated for 3 min followed by mechanical agitation for 1 hr (37 °C, 250 rpm, RT, Thermo Scientific), centrifuged (20 min, 14000 g, 20 °C, Thermo Scientific) and the clear supernatant was separated and mixed in a ratio of 1:4 with HPLC grade water. Inositol phosphates were finally eluted with 5 mL of 2 M HCl (1 mL min^−1^) using SAX column in vacuum manifold. The filtrate was evaporated under vacuum to dryness in a vacuum rotary evaporator (Hei-VAP Value Digital G3, Heidolph). The residue was re-dissolved in 1 ml of mobile phase 4.8: 5.1: 0.1 (acetonitrile: formic acid: tetrabutylammonium hydroxide, v/v/v), and subjected for Rp-HPLC (C18 RP-column, 40 °C oven temperature, 1.0 ml/min, 15 min run time).

Analytes were monitored with a PDA detector at a wavelength of 197 nm. The detector signals and/or PA peaks were processed and integrated by the chromatographic data acquisition system.

#### Transmission electron microscopy

Dormant control, T4 AS and ihp soybean seeds were allowed to imbibe water at 30 °C for 12 h. Seeds were cut into small pieces (1–2 mm) with a double-edged razor blade and fixed immediately in 2.5% glutaraldehyde buffered with 50 mM sodium phosphate, pH 7.2. Fixation was carried out at room temperature for 4 h. The tissue samples were washed four times at 15-min intervals with 50 mM phosphate buffer, pH 7.2, and post-fixed for 1 h at room temperature, with 2% aqueous osmium tetroxide. After extensive rinses in distilled water, the samples were dehydrated in a graded acetone series and infiltrated with Spurr’s resin essentially as described^35^. Thin sections were cut with a diamond knife and collected on uncoated copper grids. Sections were stained with 0.5% aqueous uranyl acetate and 0.4% aqueous lead citrate and viewed with a JEOL JEM 100B electron microscope at 100 kV.

#### Quantification of divalent metals through modified *in vivo* simulation method

To assess the bioavailability of Fe^2+^, Zn^2+^ and Ca^2+^ in T_4_ transgenic seeds, *in vitro* digestion was carried out as described by^36^. Triplicate samples weighing 5 g each were suspended in 30 ml distilled water and digested under simulated the gastrointestinal conditions, following the use of alpha amylase, gastric media containing enzymes like lipase, pepsin along with pancreatic secretions containing bile and pancreatin (Sigma Aldrich, USA). Subsequent to digestion, the suspension was centrifuged at 3600 g for 15 min. The supernatant was pooled and subjected to filtration through a filter with pore size of 0.45 μ. Distilled water was used as blank. The contents in the blank and sample was analyzed using Atomic Absorption Spectrophotometer.

Percentage of soluble mineral was calculated as bioavailability %. [Bioavailability % = Amount of Fe^2+^, Zn^+2^ and Ca ^2+^ (supernatant) – amount of Fe^2+^, Zn^+2^ and Ca ^2+^ (blank)/amount of Fe2 + , Zn^2+^ and Ca ^2+^ in undigested sample X 100].

#### Agronomic performance

The transgenic plants were subjected to agronomic evaluation under controlled conditions to study the influence of the transgene on phenotype. Mature transgenic plants were harvested and evaluated for different agronomic parameters, such as plant height, length, number of pods, number of seeds, seed dry weight, root length, stem length and dry weight. The height of individual plant was measured as the distance from the soil surface to the tip of the plant.

### Statistical analysis

The experimental data presented, are the means and standard error based on three replications. The ANOVA was implemented to compare the means, and to determine the differences in the means; chi square test was performed with P value < 0.05, assuming unequal variance.

## Results

### Generation of transgenic soybean plants

**Figure 4:**
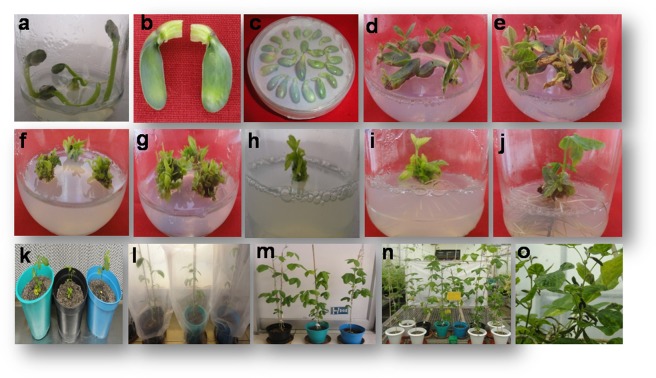
A modified cotyledonary-node method using glufosinate as the selection agent. Figure (**a**–**j**): Seedling grown in germination, shoot induction, shoot elongation medium with glufosinate selection and finally transferred into rooting medium. Figure (**k**–**o**): Plantlets were hardened in sterilized pot mix in the tissue culture facility shifted to greenhouse where plants acclimatized and reached maturity level.

**Figure 5:**
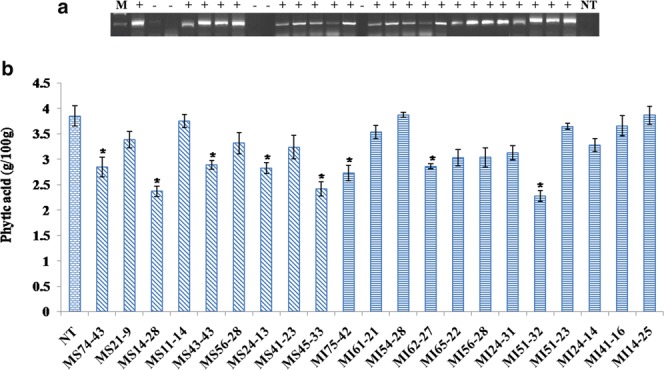
Screening of transgenic plants (**A**) using bar gene specific primers (**B**) based on phytate content. Phytate content in non-transgenic control (NT) and in T_1_ transgenic seeds were analyzed. The symbol *Indicates significant differences at p = 0.05 (n = 3).

Cotyledonary node method was used to transform *MIPS1* constructs into soybean and regenerated explants were selected on media containing different concentration of glufosinate (Fig. 4). Regenerated plantlets were transplanted and grown in a glass house (National Phytotron Facility, IARI, New Delhi). The transgenic plants so generated were screened for the presence of transgene cassette by PCR analysis using *bar* gene specific primers. The gDNA from 30-day-old plants showed amplification, whereas no amplification was observed in the non transgenic (NT) control plants. In a total of three transformation events (100 cotyledons/event), seventy-five individual soybean plants were generated of which twenty three were found positive for the transgene, as confirmed by genomic PCR analysis (Fig. 5A). Among the screened positive transgenics, five plants having MIPS As construct (T_0_; MS) and three plants with MIPS ihp construct (T_0_; MI) showing lower phytate levels significantly in the T_1_ generation (Fig. 5B). Trangenic T_1_ plants (MS14-28 and MI 51-32) exhibiting lowest phytate levels (2.32 g/100 g and 2.26 g/100 g) compared with NT (3.85 g/100 g) were further selected, and subsequent generations (T_2_–T_4_) were grown under green house conditions until maturity. After successive screening of consequent generations (T_2_–T_4_), the progeny of MS14-28 (MS14-28-12-29-3) and MI51-32 (MI51-32-22-1-13) showed low phytate levels. Thus all the analysis was performed with the progeny of these two transgenic lines in the T_4_ generation.

### Quick detection of transgenic plants

**Figure 6:**
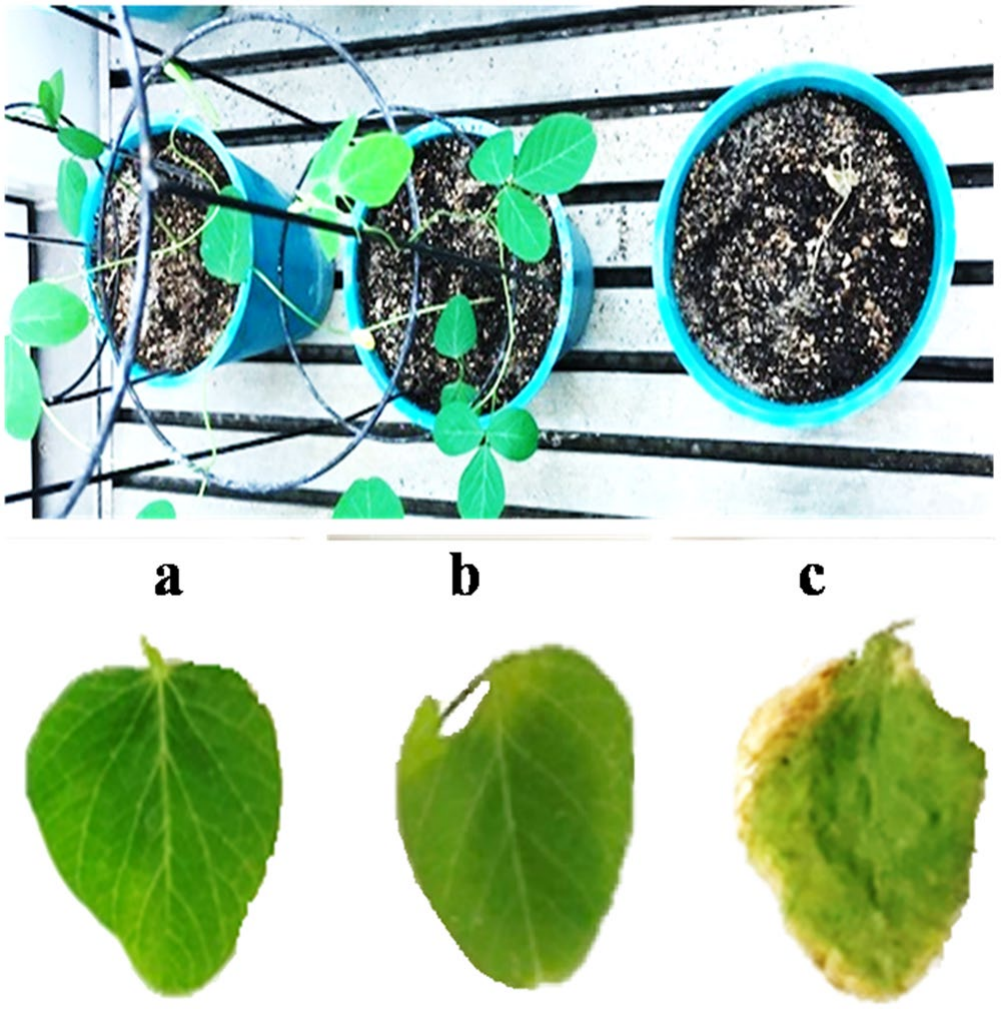
Leaf painting assay with glufosinate. Transgenic plants (**a**: AS; **b**: RNAi) showed resistance to Basta^®^ (100 mg/L) on 5^th^ days compared with NT control (**c**).

After successful transformation, large number of T_0_ transgenic plants were generated in the tissue culture lab. PCR detection of a selectable marker gene in transgenics is always a preferred choice, however there is always chance for getting false positive results. Therefore, we followed a two-step method for the screening and identification of T_0_ transgenic plants. First, screening of transformed plants was done using the *bar* gene specific primer and positive transformants were further confirmed by leaf painting assay using glufosinate (basta). After five days, painted leaves of NT control plants show lesions and turned yellow while the positive transformants leaves remained green (Fig. 6). Foliar painting/spray confirmed the tolerance to basta herbicides without any significant growth and yield effect. Thus, this method provides a low-cost, rapid, and accurate way to identify a large number of T_0_ transgenic plants.

### Expression analysis of the transgenic plants

**Figure 7:**
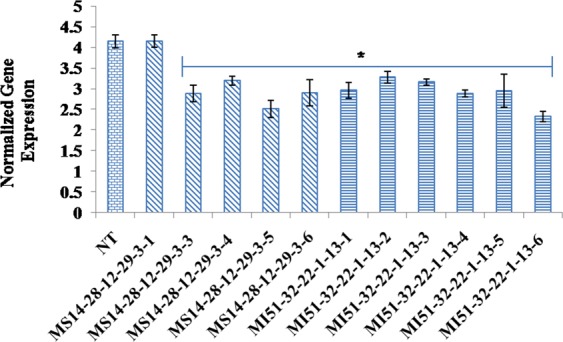
Expression analysis of transgenic rice plants. qRT-PCR analysis of T_4_ progenies of lines MS14-28-12-29-3-5, MI51-32-22-1-13-6. (NT = non-transgenic controls).

To quantify the level of down-regulation in the T_4_, MS and MI plants, quantitative real-time PCR analysis were performed using SYBR Green as a reporter dye. *PEP Carboxylase* (*PEP CO*) gene was used as the reference gene to normalize all data. The normalized expression levels of *MIPS1* transcripts varied widely among the progenies of MS14-28-12-29-3 and MI51-32-22-1-13 (Fig. 7). However, a maximum normalized reduction of 1.8 times was observed in the transgenic line MS14-28-12-29-3-5 (T_4_) among MIPS As progenies and 1.9 times in MI51-32-22-1-13-6 (T_4_) among MIPS ihp progenies, revealing a distinct knock down of *MIPS1* gene. Comparative expression analyses of T_4_ transgenics also underlines the facts that RNAi mediated silencing was better compared to antisense for successful down regulation of *MIPS1* gene.

### Southern blot analysis of the transgenic plants

**Figure 8:**
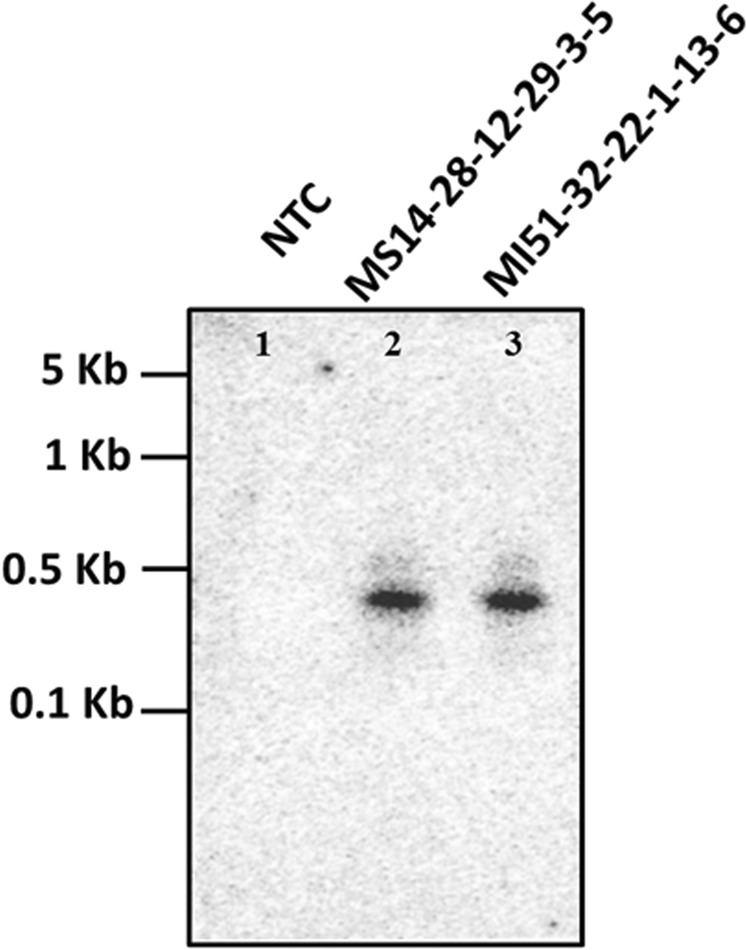
Southern blot analysis of T_4_ progenies (MS14-28-12-29-3-5 and MI51-32-22-1-13-6). Each line consist of 10 μg genomic DNA, digested with *Xho*I. The position and sizes of markers are indicated (NTC = Non-transgenic control).

The integration of transgene cassette using *bar* gene specific probe in the genome of lines MS14-28-12-29-3-5 and MI51-32-22-1-13-6 were confirmed by southern blot analyses using *Xho*I (Fig. 8). The results revealed the stable integration of transgene cassette into the T_4_ progenies. The expected ~500 bp bands were observed in both lines; however, no hybridization signal was detected for the NT control NTC plants.

### Seed phytic acid contents in transgenic soybean lines

**Figure 9:**
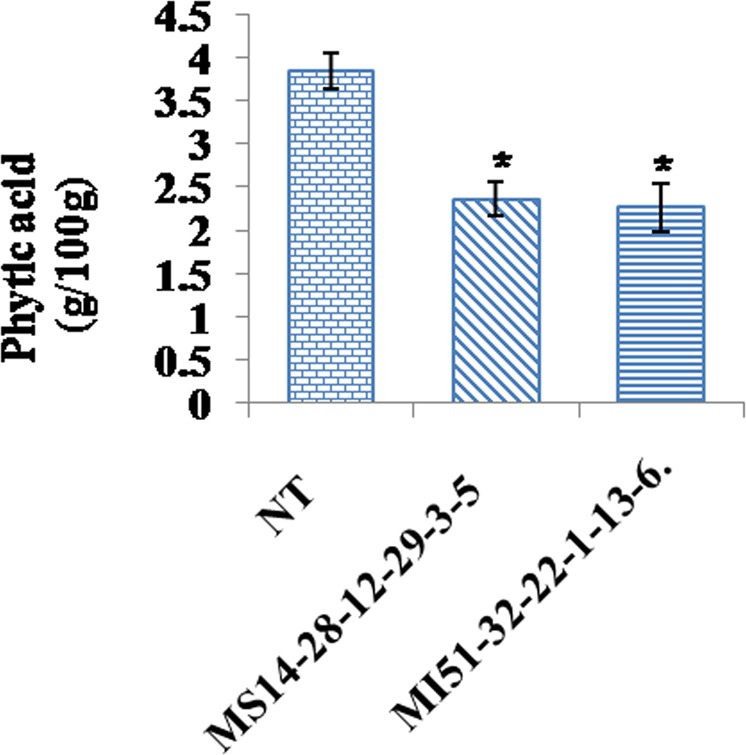
Analysis of seed phytate content in non transgenic control (NTC) and transgenics (T_4_). Phytate content of transgenic seeds (MS14-28-12-29-3-5 and MI51-32-22-1-13-6) compared to NTC seeds showed significant differences (P < 0.05).

In order to assess the level of phytate reduction quantitatively in T_4_ transgenic seeds, HPLC analysis was carried out. The chromatogram obtained from HPLC/UV–VIS method, at 197 nm suggested a significant decrease in the levels of PA in the transgenic lines (MS14-28-12-29-3-5 and MI51-32-22-1-13-6) as compared to the NT control. The mean PA values, as calculated from the corresponding peak area, were 3.87 ± 0.13 5 g/100 g for NT seeds, 2.37 ± 0.13 for MS14-28-12-29-3-5 and 2.27 ± 0.05 for MI51-32-22-1-13-6 (Fig. 9). These results represented an average reduction in the seed phytate content of 38.75% for line MS14-28-12-29-3-5 and 41.34% for line MI51-32-22-1-13-6.

### Ultrastructural analysis of seed phytate content by electron microscopy

**Figure 10:**
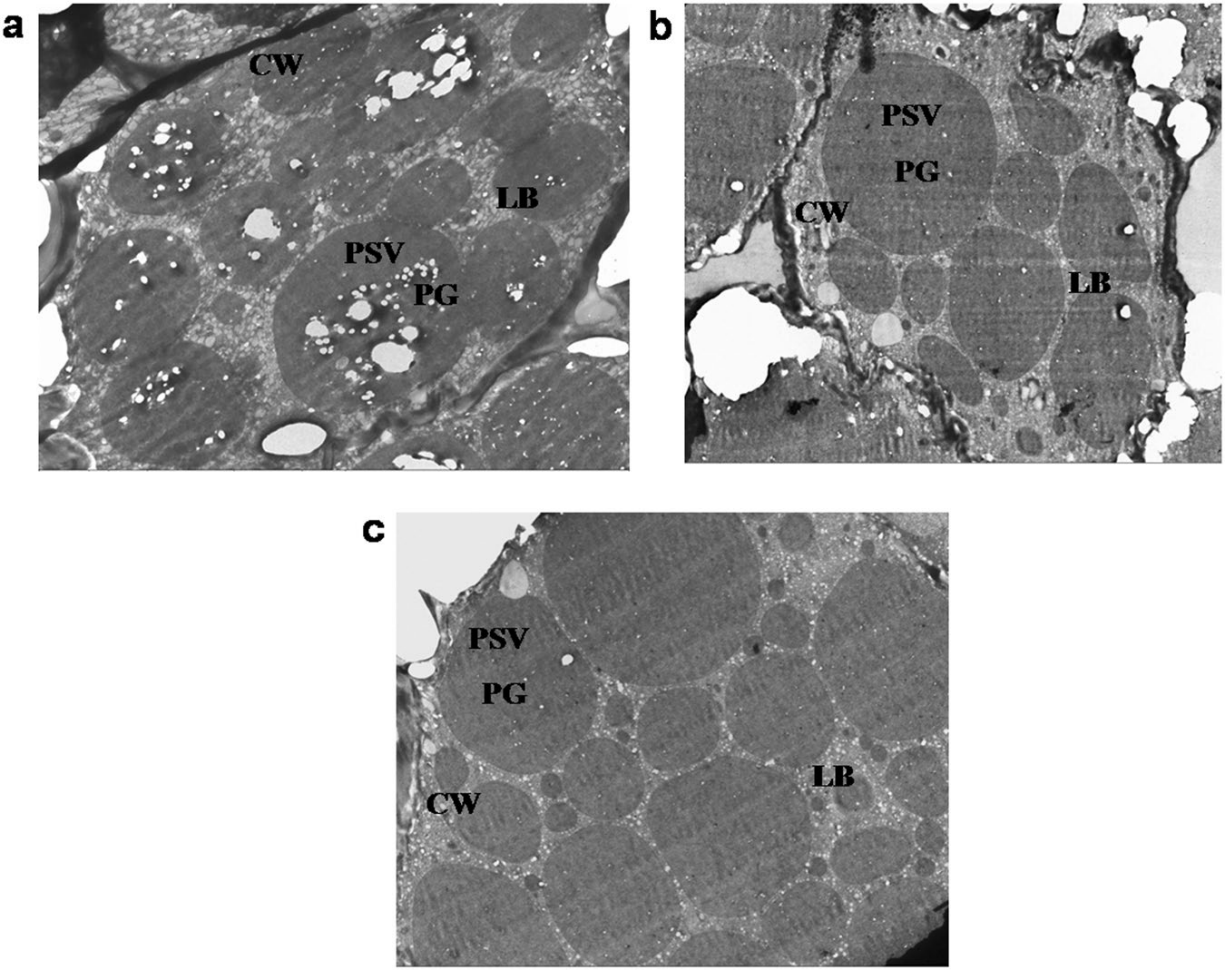
Electron microscopic analysis of NT and transgenic (T_4_) seeds. (**a**) Cotyledon sections of NTC (**b**) MS14-28-12-29-3-5 (**c**) MI51-32-22-1-13-6 lines. Phytin globoid (PG) cavities are the small white circular areas. (PSV-Protein storage vacoules; CW- Cell wall; LB-lipid bodies).

Qualitative analysis of phytate content was assessed by electron microscopy. As in soybean seeds, PA is complexed with mineral salts (phytins) and located within PSVs. The electron dense globoids can be visualized by transmission electron microscopy. Transmission electron micrographs of PSVs in the cotyledons of NT control revealed the presence of numerous globoid phytate crystals. When seeds are subjected to conventional chemical fixation, water-solubilized phytin deposits in the globoid crystals are not well preserved, resulting in the appearance of cavities. Globoid crystals (cavities) in the PSVs of mature T_4_ seeds of MS14-28-12-29-3-5 and MI51-32-22-1-13-6 were significantly less frequent than those of non-transformed seed (Fig. 10).

### Bioavailability of divalent metals (Fe^2+^, Zn^2+^ and Ca^2+^)

**Figure 11:**
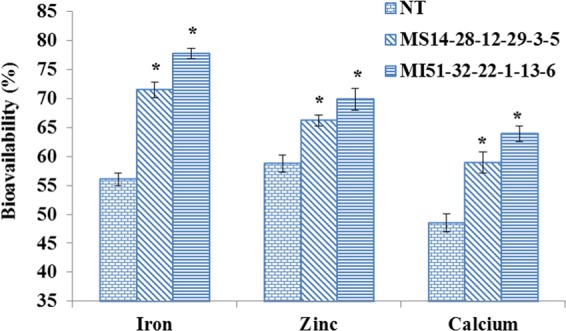
Bioavailability of metal ions in NT and transgenics (T_4_). Bioavailability of divalent minerals (Fe^2+^, Zn^2+^ and Ca^2+^) using *in vivo* simulation model showed significant (P < 0.05) increase in Transgenics (MS14-28-12-29-3-5 and MI51-32-22-1-13-6) compared to NT control.

PA is well known as a potent chelator of essential minerals and therefore, renders the divalent ions unavailable. Hence, we examined the *in vivo* bioavailability of Fe^2+^, Zn^2+^ and Ca^2+^ in low phytate T_4_ transgenic seeds compared to that of NT control using atomic absorption spectrophotometer. The bioavailability was found to be higher in both transgenic T4 lines, i.e. MS14-28-12-29-3-5 and MI51-32-22-1-13-6 compared to controls (Fig. 11). Among the lines, as expected MI51-32-22-1-13-6 was found to be better with bioavailability of 21.7% Fe^2+^, 11.15% Zn^2+^ and 15.35% Ca^2+^ compared to NT control. Among the different metals analyzed, iron bioavailability increased to a maximum of 77.75% and 71.45% in T_4_ lines compared to 56.05% in NT control. MS14-28-12-29-3-5 line also showed an increase of 15.4% (Fe^2+^), 7.45% (Zn^2+^) and 10.4% (Ca^2+^) compared to NT control.

### Agronomic performance of transgenic plants

**Figure 12:**
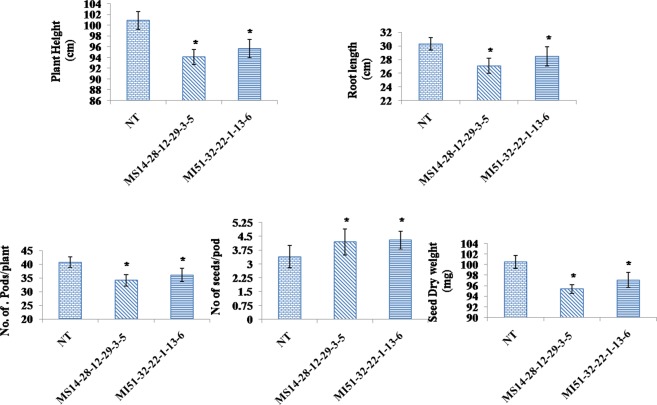
Various parameters considered for agronomic evaluation of T_4_ transgenic plants grown in green house. Values are mean ± SE, n = 10 (P ≤ 0.002).

The transgenic lines and the un-transformed control were subjected to agronomic evaluation under control condition to study the seed specific silencing of transgene of the phenotype (Fig. 12). Significant variation in the major agronomic traits was observed without much detrimental effects (P ≤ 0.002). Both transgenic lines exhibited shorter height, root length and number of pods/plant compared to control (Fig. 12). At mature stage, normally filled grains (full seeds) were observed slightly more in transgenics with lesser dry weight.

## Discussion

Phytic acid, the major phosphorous reserve observed to increase steadily during seed development until maturation, act majorly as a chelator of essential minerals. The present study demonstrates an efficient reduction in the PA content using AS and RNAi constructs driven under the control of a seed specific promoter, designed for down-regulation of *MIPS1* isoform, emphasizing its role in PA accumulation in soybean seeds. Phosphate starvation studies have evidently proved that phytate is not a prime requirement for seed viability or germination and can be limited to certain levels in seeds for better mineral bioavailability^37,38^. Metabolic engineering of phytate biosynthetic pathway offers a valuable option for manipulating an effective gene target for successfully paving a path towards low phytate soybean. Down-regulation of gene expression via AS or RNAi constructs at post transcriptional levels has been widely reported since past two decades, not only to develop mutants but also for functional characterization of the genes. In the present study, we have employed and compared both the post transcriptional gene silencing (PTGS) approaches (AS and RNAi) to down regulate the expression of the *MIPS1* gene, encoding the enzyme catalyzing the first step of the phytate biosynthesis pathway. Transcriptional profiling of *MIPS* isoforms in soybean had revealed *MIPS1* to be the highest expressing isoform in developing seeds^25^, which makes it a potential target for gene silencing. The expression studies were in conformity with the earlier reports^39^. MIPS being the rate limiting enzyme, where PA is synthesized from glucose-6-phosphate, low phytate mutants targeting *MIPS* have been developed in various crops^2,4,12,40^. Earlier reports on down regulation of *MIPS* activity under constitutive *CaMV35S* promoter via AS RNA not only presented a reduction of 7% in inositol content but also showed smaller tubers, altered leaf morphology, precocious leaf senescence and reduced apical dominance^14,41^. *MIPS* expression under *Ole18* promoter, however, directed the expression specifically in the aleurone layer and embryos of the rice seeds^19,42^, thereby, generating viable mutants with 58% decline in phytate content in rice^18^. Similarly in soybean, PA reduction up to 94.5% through RNAi induced down-regulation of *MIPS1* under the constitutive *CaMV35S* promoter was reported, but drastic reduction of *myo-*inositol phosphates - intermediates in the phytate biosynthesis pathway, being precursors in various metabolic pathways involved in signal transduction, stress response, membrane biogenesis and auxin physiology *etc* probably led to sterile transgenic seeds or transgenics with poor phenology^17^.

In this study, we used a modified version of cotyledonary node method for soybean transformation having better transformation efficiency^43^. The transgenic plants were thus generated, where *MIPS1* gene expression was manipulated tissue specifically through the use of *vicilin* promoter by AS as well as RNAi approaches. The transgenic plants showed stable integration of the transgene cassette and displayed a normal phenotype. After initial PCR screening, leaf painting assay was employed (basta @ 100 mg/L) for identification and confirmation of the positive T_0_ transgenic plants^28,29^. Leaf painting directly detected the effect of a selectable marker gene and overcame false positives obtained by PCR screening. More so, being low-cost and easy to conduct, it was a quick and effective method to identify the positive transformants from amongst a large number of T_0_ plants generated as it was critical for subsequent management of the plantlets.

In transgenic seeds, the normalized fold expression of *MIPS1* at transcriptional level suggested effective silencing with 1.8–1.9 times suppression of *MIPS1* compared to NT control in MS14-28-12-29-3-5 and MI51-32-22-1-13-6 T_4_ lines respectively. In view of previous studies, an obvious implication of silencing MIPS is the decrease in phytate levels^9^ which was confirmed when the T_4_ generation seeds of line MS14-28-12-29-3-5 and MI51-32-22-1-13-6 showed a 38.75% and 41.34% decline in the amount of PA as compared to NT control. Since the reduction in PA levels were not drastic, the aberrations like seed abortions, loss of germination or other agronomic performance abnormalities could probably not be observed in the developed lines.

In soybean plants, each essential mineral displays a characteristic accumulation pattern. These metal ions are translocated to seeds via xylem and phloem through various transporters viz. ZIPS, IRT, YSL, etc., and the mineral content of harvested seeds impact food and feed quality. Most of these divalent cations are chelated by PA, which reduces the mineral bioavailability in non-ruminants^44,45^, probably due to lack of phytase enzyme in them^26^. In view of these assumptions, we analyzed the bioavailability of Fe/Ca/Zn using an *in vivo* mimicking model^33^ and observed a significant increase in the minerals bioavailability in MS14-28-12-29-3-5 and MI51-32-22-1-13-6 lines compared with NT control. An increase in the mineral content in low phyate lines has also been reported in rice^18^ and wheat^46^.

Post synthesis, PA is transported to the protein storage vacuoles and stored as phytate globoids. The site of PA accumulation however differs from species to species. In rice and wheat, the phytate globoids are predominant in the aleurone layer^18,46^ and in maize in the embryo^47^. In case of soybean, however, the protodermal cells of developing seeds form the major site of PA accumulation^17^. The Transmission Electron Microscopy studies conducted in the mature T_4_ seeds of the AS and RNAi transgenic lines showed a reduction in the number of the phytate globoids compared to the non transformant, confirming the down-regulation of *MIPS1*. Previously, a reduction in the phytate globoids in low phytate lines was also reported^17,48^.

Agronomic traits studied in the T_4_ transgenics revealed a significant variation in the without much detrimental effects. Plant heights which serve as a general indicator of plant vigor were found to be decreased and might be possibly due to change in intermodal cell length by the role of inositol polyphosphate phosphatases^49^. The phenotypic effect observed on the root growth precludes the possibility of an altered phosphate homeostasis in transgenic lines. The freely available phosphorus limits the need for sensing phosphorus and this reduce the root architecture. In line with our observation Paulik *et al*.^50^ has observed decreased surface area of roots, alter length of root hairs and slower elongation rates in low phytate IPK1 mutants. The deregulation in the phosphate accusation machinery internal affected by various inositol intermediates playing major role as signaling molecules might be the possible reason for the observed decrease in the agronomic traits. There is a common tradeoff between seed number and weight and it was well evident in transgenics compared to control (Supplementary Fig. 3).

Evidences presented in this study signifies that the viable transgenics with moderate reduction in phytate levels and improved mineral bioavailability was resulted due to seed specific silencing of GmMIPS1.

## Conclusion

The results obtained in the present study thus demonstrated that RNAi and AS mediated silencing of *MIPS1* gene expression under seed specific *vicilin* promoter were effective strategies to reduce the phytate levels and improve the mineral bioavailability in soybean seeds and that *MIPS1* could be recommended as a potential target for manipulating the PA pathway without any detrimental effects on other cellular, molecular and physiological processes.

## Supplementary information

Suuplemntary figures

## Data Availability

The datasets generated during and analyzed during the current study are available from the corresponding author on reasonable request.
