# Supplementary material for: Seed targeted RNAi-mediated silencing of GmMIPS1 limits phytate accumulation and improves mineral bioavailability in soybean
Source: Sci Rep. 2019 May 23;9:7744. doi: 10.1038/s41598-019-44255-7 (PMC6533290; doi:10.1038/s41598-019-44255-7)
Supplement: Supplementary file 1 — Suuplemntary figures [file 41598_2019_44255_MOESM1_ESM.pdf]

Supplementary information

Seed targeted RNAi-mediated silencing of *GmMIPS1* limits phytate accumulation and improves mineral bioavailability in soybean.

Awadhesh Kumar[1,2], Varun Kumar[1,3], Veda Krishnan[1], Alkesh Hada[1], Ashish Marathe[1], Parameswaran C[1,2], Monica Jolly[1] & Archana Sachdev[1*]

Division of Biochemistry, ICAR-Indian Agricultural Research Institute, New Delhi- 110 012, India[1]

Division of Crop Physiology and Biochemistry, ICAR-National Rice Research Institute, Cuttack, Odisha, India[2]

Department of Biotechnology and Bioinformatics, Jaypee University of Information Technology, Waknaghat (H.P.), India[3]

***Corresponding Author:**

Dr. Archana Sachdev
Division of Biochemistry,
Indian Agricultural Research Institute,
New Delhi- 110 012, India
E mail: arcs_bio@yahoo.com
Tel. No: +91-9810683014

Supplementary figures:

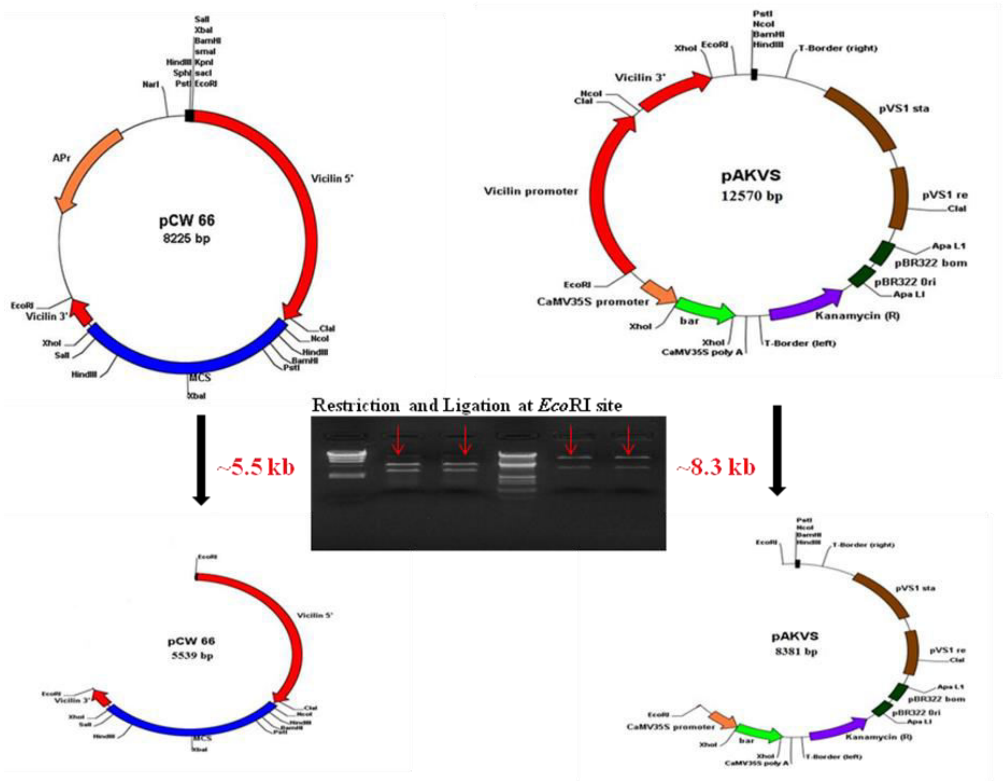

Supplementary Fig1. Circular maps of pCW66 and pAKVS vectors. Restriction and ligation of pCW66 + pAKVS fragment (~5.5 kb + ~8.3 kb) at *Eco*RI site to generate a new binary vector pCWAK. 1% agarose gel showing *Eco*RI restricted fragment of both vectors along with λ *Hin*dIII and λ *Eco*RI/*Hin*dIII markers.

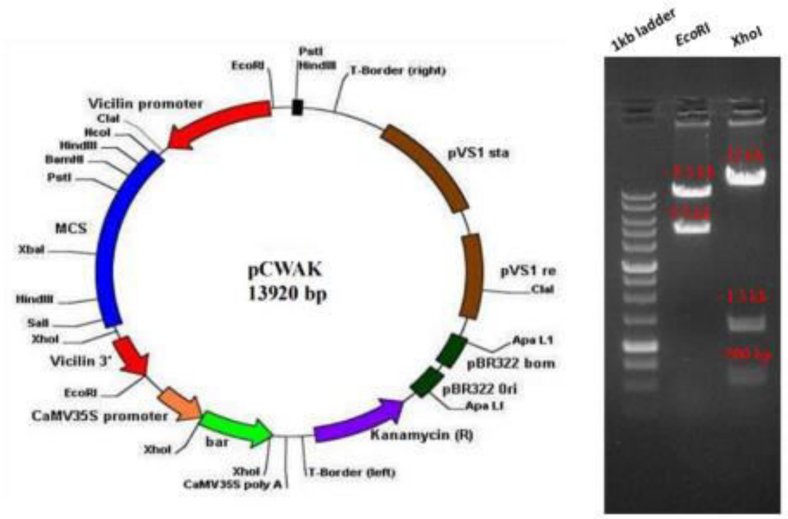

Supplementary Fig 2. Circular map of new binary vector pCWAK containing vicilin promoter with multiple restriction sites, plant selection marker *bar* gene and bacterial selection marker *kanamycin*. Restriction digestion of pCWAK; Lane 1: 1 kb ladder, lane 2: *Eco*RI digested pCWAK showing upper band of pAKVS (8.3 kb) and lower band of pCW66 (5.5 kb), Lane 3: *Xho*I digested pCWAK showing lower band of *bar* gene (500 bp).

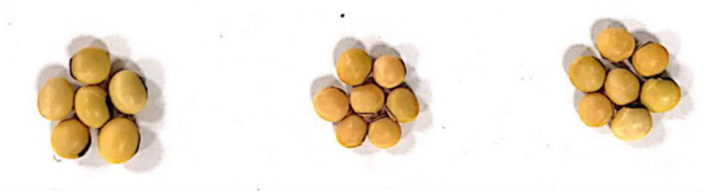

NT **MS14-28-12-29-3-5** **MI51-32-22-1-13-6**

Supplementary Fig 3. The transgenic lines and the non-transformed control were subjected to agronomic evaluation under control condition. Mature seeds in the transgenic lines were all viable, though per gram dry weight is slightly low but are more in number.
